# Surface Integrity and Corrosion Resistance of 42CrMo4 High-Strength Steel Strengthened by Hard Turning

**DOI:** 10.3390/ma14226995

**Published:** 2021-11-18

**Authors:** Qingzhong Xu, Yan Liu, Haiyang Lu, Jichen Liu, Gangjun Cai

**Affiliations:** 1 Naval Architecture and Ocean Engineering College, Dalian Maritime University, Dalian 116026, China; ly_dmu@163.com (Y.L.); liujichen@dlmu.edu.cn (J.L.); caigangjun97@dlmu.edu.cn (G.C.); 2 Polar Ship Navigation Safety Research Center, Dalian Maritime University, Dalian 116026, China; 3 Key Laboratory of High Efficiency and Clean Mechanical Manufacture of MOE, School of Mechanical Engineering, Shandong University, Jinan 250061, China; luhaiyang2011@126.com

**Keywords:** hard cutting, surface integrity, corrosion resistance, 42CrMo4 steel

## Abstract

To improve the surface corrosion resistance of 42CrMo4 high-strength steel used in a marine environment, this article studied the effects of hard turning on the surface integrity and corrosion resistance of 42CrMo4 high-strength steel through the single factor experimental method, namely hard turning, polarization corrosion, electrochemical impedance spectroscopy, potentiodynamic polarization curve, and salt spray tests. The results indicated that the surface integrity was modified by the hard turning, with a surface roughness lower than *Ra* 0.8 μm, decreased surface microhardness, fine and uniform surface microstructure, and dominant surface residual compressive stress. The hard turning process was feasible to strengthen the surface corrosion resistance of 42CrMo4 high-strength steel. The better corrosion resistance of the surface layer than that of the substrate material can be ascribed to the uniform carbides and compact microstructure. The corrosion resistance varied with cutting speeds as a result of the changed surface microhardness and residual compressive stress, varied with feed rates as a result of the changed surface roughness, and varied with cutting depths as a result of the changed surface residual compressive stress, respectively. The surface integrity with smaller surface roughness and microhardness and bigger surface residual compressive stress was beneficial for corrosion resistance.

## 1. Introduction

As is known, hard machining has been widely used in the machine finishing of hardened steels [1,2,3]. With the development of high-performance cutting tools, a more stable hard cutting process and higher machining efficiency can be obtained [4,5]. However, the modification of workpiece surface integrity in the matter of surface roughness, microhardness, microstructure, and residual stress is difficult to predict, owing to the existence of the cutting zone under high temperature and pressure in the process of hard cutting. Moreover, surface integrity has a significant impact on corrosion resistance, which directly affects the service life of parts [6,7]. Currently, 42CrMo4 high-strength steel has been extensively used in the marine industry, such as for shaft parts, crankshaft, connecting rod, drilling joint, pump parts, steam turbine, and salvage equipment [8,9]. In order to ensure performance reliability in the marine environment, excellent corrosion resistance of machined surfaces for components and parts made of 42CrMo4 high-strength steels is essential. Therefore, it is important to study the influences of hard cutting on the surface integrity and corrosion resistance of 42CrMo4 high-strength steel and reveal the inherent relation between surface integrity and corrosion resistance.

Some scholars have conducted research on the surface characteristics and corrosion behaviours of machined metal surfaces [10,11,12,13,14]. Rajaguru et al. [15] demonstrated that the ferrite phase of super duplex stainless steel was easier to suffer stress corrosion cracking behaviour than the austenite phase in the chloride environment. Wan et al. [16] found that residual stress and micro-cracks had a significant impact on the corrosion resistance of machined aluminum alloy parts. Zhang et al. [17] reported that in the hard turning and low plasticity burnishing of Cr-Ni alloys, residual compressive stress and surface roughness were more important than grain refinement and microhardness in improving corrosion resistance. Liu et al. [18] pointed out that the thick oxide film on the machined surface produced in the high-speed dry cutting of 17-4PH stainless steel was conducive to corrosion resistance. Su et al. [19] demonstrated that the surface finishing after ultrasonic roller burnishing was related to the corrosion resistance of TC11 titanium alloy. Kumar et al. [20] pointed out that the grain refinement and compressive residual stresses induced by ultrasonic shot peening can strengthen the corrosion resistance of Ti-13Nb-13Zr alloy. However, the studies on the effects of hard cutting on the surface integrity and corrosion resistance of 42CrMo4 high-strength steel are still insufficient, and the inherent relation between surface integrity and corrosion resistance needs deep research.

In the present study, the hard turning process was adopted to strengthen the surface integrity and corrosion resistance of 42CrMo4 high-strength steel and the inherent relation between surface integrity and corrosion resistance was revealed. This work can guide the practical production and application of the hard cutting process in strengthening the surface integrity and corrosion resistance of 42CrMo4 high-strength steel.

## 2. Materials and Methods

### 2.1. Hard Turning Experiment

The workpiece was 42CrMo4 high-strength steel bar (Hunan Valin Xiangtan Iron and Steel Co., Ltd., Hunan, China) with a size of *Ø*70 × 300 mm, and the macrohardness of the steel bar was 50 HRC after the heat treatment of water quenching at 850 °C and low temperature tempering at 200 °C. The chemical compositions of 42CrMo4 high-strength steel are listed in Table 1. The continuous hard turning experiment without cutting fluid was carried out on a CA6140A lathe (Shenyang Machine Tool Co., Ltd., Shenyang, China). The BT6000 CBN tool with an ISO designation of CNGA 120412 (Zhengzhou Berlt Hard Materials Co., Ltd., Zhengzhou, China) was used, and its geometric parameters on the cutter bar are listed in Table 2. To eliminate the disturbing of tool wear, the new cutting edge was applied in each turning experiment.

**Figure 1:**
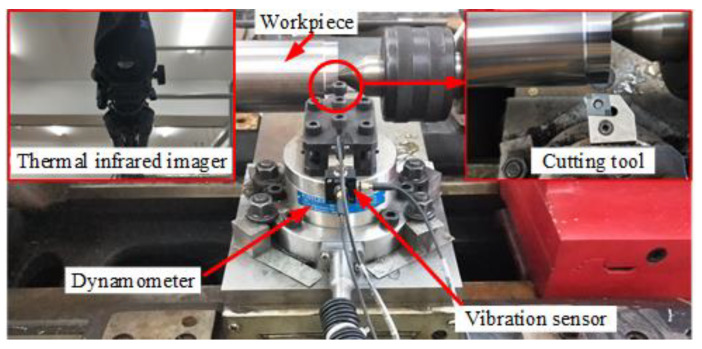
Photographic view of hard turning experiment.

Table 3 shows the single factor experimental method to research the effects of cutting parameters on the surface integrity and corrosion resistance of 42CrMo4 high-strength steel. Experiments #1, #2, #3, and #4 were used to investigate the effects of cutting speed, Experiments #2, #5, #6, and #7 were used to investigate the effects of feed rate, and Experiments #8, #2, #9, and #10 were used to investigate the effects of cutting depth. Figure 1 presents the photographic view of hard turning experiment. In the initial cutting stage, the cutting force, cutting temperature, cutting vibration, and surface roughness were obtained with a dynamometer (Model 9272, Kistler Group, Winterthur, Switzerland), a thermal infrared imager (Model A325SC, Teledyne FLIR, Wilson Ville, OR, USA), an acceleration acquisition system (Model DH5922N, Jiangsu Donghua Testing Technology Co., Ltd., Jiangsu, China), and a roughness meter (Model 3200, TIME Group Inc., Beijing, China), respectively. In the roughness measurement, the Gaussian filter was used with an evaluation length of 4 mm and a cutoff value of 0.8 mm [21]. In order to ensure the accuracy of test data, five repeated tests were carried out under each group of turning parameters.

### 2.2. Finite Element (FE) Model

**Figure 2:**
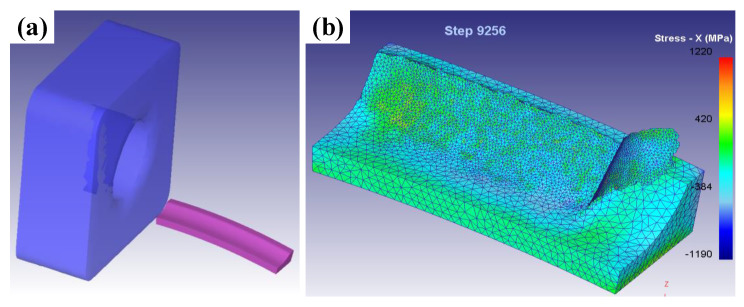
(**a**) Model of tool and workpiece and (**b**) residual stress distribution.

In this study, the surface residual stress was obtained by the FE method using the Deform 3D software. The cutting tool was modeled by SolidWorks software. The workpiece was set to AISI4140 steel with the hardness of 50 HRC. The model of tool and workpiece are shown in Figure 2a. The adaptive tetrahedral mesh was adopted, and the amounts of tool mesh was 25,000. The minimum mesh size of the workpiece was set to 40% feed rate. The environment temperature was 20 °C, and the heat transfer coefficient was 0.02 N/s/mm/C. When the simulation was finished after 10,000 steps, the workpiece was cooled in air to environment temperature with the heat transfer coefficient of 100 N/s/mm/C. Then, the surface residual stress was obtained as shown in Figure 2b. The accuracy of the simulation model was validated in terms of cutting forces with experimental results.

### 2.3. Sample Preparation and Characterization

All the samples used in the following tests were cut from the workpiece bar and treated by ultrasonic cleaning in acetone. According to the requirement of each test, the rough grinding, fine grinding, and polishing treatments were chosen for the corresponding surfaces of samples.

The microhardness value was the mean of three measurements under a load of 100 g for 10 s using a digital microhardness tester (Model HV-1000, Precision Meter (Dongguan) Co., Ltd., Dongguan, China). To visually demonstrate the effect of surface roughness on the corrosion resistance of 42CrMo4 steel, the surface topographies of samples obtained in Experiments #5 and #6 were observed with a 3D surface profiler (Model MicroXAM, KLA Corporation, Milpitas, CA, USA). The microstructures were investigated by a metallurgical microscope (Model DMI8C, Leica Microsystems Inc., Buffalo Grove, IL, USA) and a scanning electron microscope (SEM) (Model Quanta 250 FEG, FEI Company, Hillsboro, OR, USA), respectively.

### 2.4. Electrochemical Experiment

In this study, to investigate the effect of hard turning on the corrosion resistance of 42CrMo4 high-strength steel, the electrochemical test and salt spray test were applied, respectively. The electrochemical test was carried out on an electrochemical workstation (Model P3000A, AMETEK Inc., Berwyn, PA, USA) at room temperature (20 ± 2 °C) with the sample electrode as working electrode, saturated calomel electrode as reference electrode, platinum net as counter electrode, and 3.5 wt.% NaCl solution as electrolyte. The polarization corrosion test was conducted on the sample cross section perpendicular to the feed direction. The size of the cross section was 10 mm × 5 mm, and the polarization corrosion test was performed in a potential range of −1 V to 0 V at a scanning rate of 1 mV/s. After the polarization corrosion test, the corrosion morphology of the sample was observed by the scanning electron microscope. The electrochemical impedance spectroscopy (EIS) and potentiodynamic polarization curve tests were carried out on the machined sample surfaces, and the size of the test area was 10 mm × 10 mm. The EIS test was conducted in a frequency span from 100 kHz down to 0.01 Hz with an amplitude of 10 mV. The potentiodynamic polarization curve test was conducted in a potential range of −1.2 V to 0.2 V at a scanning rate of 1 mV/s. The corrosion potential (*E*_corr_) and current density (*I*_corr_) were obtained by the Tafel-type fit analysis software installed in the electrochemical workstation.

The salt spray test for the machined sample surfaces was conducted in a corrosion test chamber (Model SFMIT-40A, Changzhou Sanfeng Instrument Technology Co., Ltd., Changzhou, China) at 35 ± 2 °C with the 5 wt.% NaCl neutral solution. The size of the test area was 20 mm × 30 mm, and the salt spray sedimentation rate was 1–2 mL/(80 cm^2^·h). After the salt spray test, the corrosion products on samples were removed according to the GB/T 16545-2015 standard. Following the measurement of sample weight loss, the annual corrosion rate was calculated by the below equation:

(1) C.R.=(87600×W)/(S×t×D)

where *C.R.* is the annual corrosion rate (mm/a), *W* is the sample weight loss (g), *S* is the sample surface area (cm^2^), *t* is the corrosion cycle (h), and *D* is the steel density (g/cm^3^). For each group of samples, three tests were carried out to ensure data reproducibility.

## 3. Results and Discussions

### 3.1. Effect of Hard Turning on the Surface Integrity

#### 3.1.1. Surface Roughness

**Figure 3:**
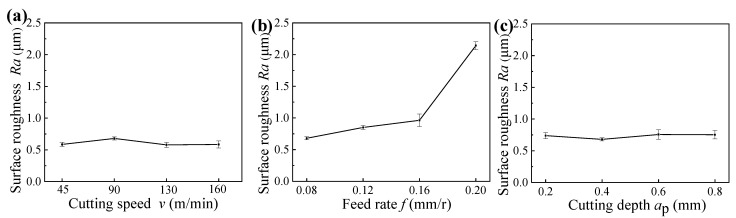
Surface roughness of samples machined under different cutting parameters: (**a**) cutting speed, (**b**) feed rate, and (**c**) cutting depth.

Figure 3 shows the surface roughness of machined samples under different cutting parameters. It can be found that the feed rate affected the surface roughness greatly, whereas the cutting speed and cutting depth had slight effects on the surface roughness. When the feed rate was smaller than 0.12 mm/r, the surface roughness obtained under different cutting speeds and cutting depths were all lower than *Ra* 0.8 μm, and this indicates that the hard turning can obtain the same surface roughness as finish grinding.

**Figure 4:**
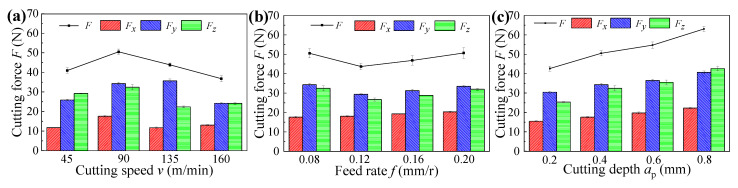
Cutting forces obtained under different cutting parameters: (**a**) cutting speed, (**b**) feed rate, and (**c**) cutting depth.

**Figure 5:**
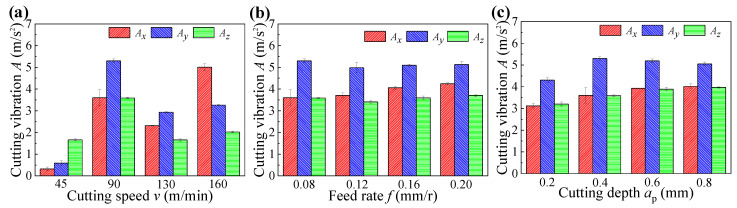
Cutting vibrations obtained under different cutting parameters: (**a**) cutting speed, (**b**) feed rate, and (**c**) cutting depth.

Figure 4 indicates the cutting forces under different cutting parameters, in which the *F*, *F_x_*, *F*_y_, and *F_z_* mean the resultant cutting forces, axial forces, radial forces, and tangential forces, respectively. Figure 5 indicates the cutting vibrations under different cutting parameters, in which the *A*_x_, *A*_y_, and *A_z_* mean the axial vibrations, radial vibrations, and tangential vibrations, respectively. As shown in Figure 4 and Figure 5, the cutting vibrations were associated with the cutting forces. The radial forces were the dominant cutting force component, and caused big cutting vibrations in the radial direction. The different radial vibrations were responsible for the different surface roughness of machined samples obtained under different cutting speeds and cutting depth. As a result of decreased cutting forces and cutting vibrations, the surface roughness of machined samples decreased with increased cutting speeds, whereas the surface roughness of machined samples presented the opposite trend with increased cutting depths, resulting from increased cutting forces and cutting vibrations.

#### 3.1.2. Microhardness

**Figure 6:**
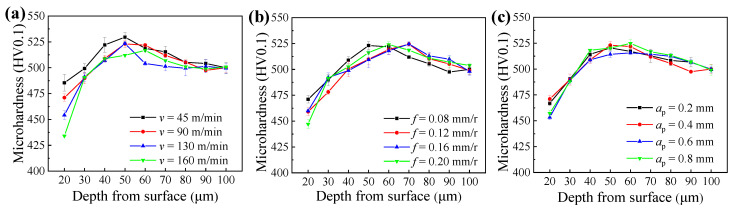
Microhardness of samples machined under different cutting parameters: (**a**) cutting speed, (**b**) feed rate, and (**c**) cutting depth.

Figure 6 shows the microhardness distribution of machined samples under different cutting parameters, and each sample presented an approximate trend of microhardness gradient. From the machined surface to the substrate, the microhardness increased first, then decreased, and finally stabilized at the depth of 100 μm. Furthermore, according to the microhardness gradient, the area 0–25 μm away from the machined surface is labeled as the surface layer, the area 25–100 μm away from the machined surface is labeled as the subsurface layer, and the area over 100 μm away from the machined surface is labeled as the substrate material. The microhardness of the surface layer was lower than that of the substrate, whereas the subsurface layer presented the highest microhardness, and the highest microhardness of the subsurface layer was 23 ± 4 HV0.1 higher than that of the substrate.

**Figure 7:**
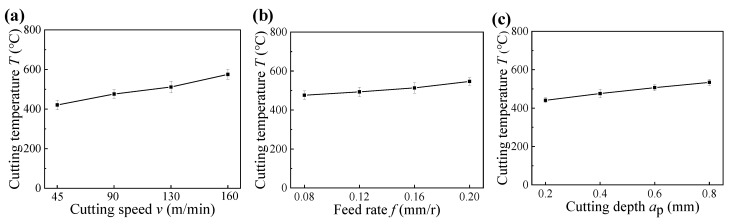
Cutting temperatures obtained under different cutting parameters: (**a**) cutting speed, (**b**) feed rate, and (**c**) cutting depth.

The formation of microhardness gradient was determined by the grain deformation resulted from cutting force, and the thermal effect resulted from cutting temperature [22]. Figure 7 presents the cutting temperatures under different cutting parameters, respectively. Due to the small cutting force between 35 N and 65 N (as illustrated in Figure 4) and the high cutting temperature between 400 °C and 600 °C (as shown in Figure 7) in the hard cutting, the microhardness of the machined sample was mainly influenced by the cutting temperature. Thus, the surface layer suffered secondary tempering and presented decreased microhardness owing to the softening effect [23]. In addition, due to the big influence of cutting speed on the cutting temperature (as shown in Figure 7a), the influence of cutting speed on the microhardness of the surface layer was bigger than those of the feed rate and cutting depth, and the softening degree of the surface layer increased with the increase in cutting speed (as illustrated in Figure 6a). Furthermore, the higher microhardness of subsurface layer can be attributed to the plastic deformation caused by the radial forces (as illustrated in Figure 4), and the highest microhardness of subsurface layer was directly proportional to the radial forces.

#### 3.1.3. Microstructure

**Figure 8:**
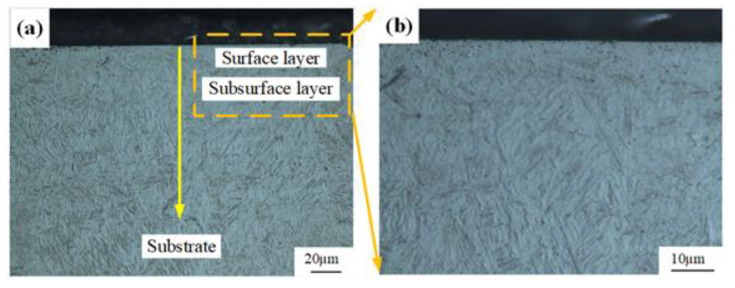
Metallographic structures of machined sample: (**a**) overall view and (**b**) local enlarged view.

**Figure 9:**
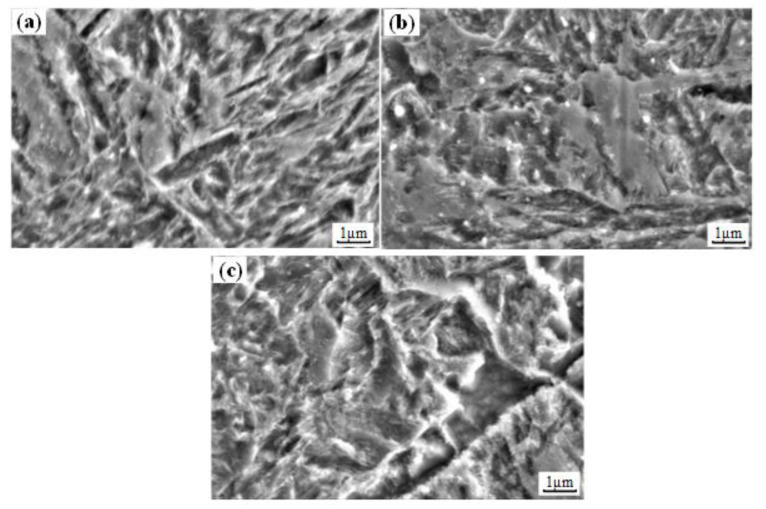
SEM morphologies of (**a**) surface layer, (**b**) subsurface layer, and (**c**) substrate.

Figure 8 shows the metallographic structures of the machined sample obtained in Experiment #2, and the microstructures from the surface layer to the substrate were mainly tempered martensites. In the surface layer, due to the thermo-mechanical coupling effects, the morphology of martensite was not obvious (as shown in Figure 8b). In the subsurface layer and substrate, the martensite presented lath-like morphology. To further analyze the influence of hard cutting on the microstructures of the machined sample, the SEM observation were applied. Figure 9 displays the SEM morphologies of the machined sample.

The microstructure of the surface layer was fine and uniform, whereas the subsurface layer presented coarse and uneven microstructure, and the microstructure of the substrate material was moderate. This can be explained by the precipitation of carbides [23,24,25]. Under the effect of high cutting temperature, the diffusion ability of elements in the surface layer was enhanced, then the carbides precipitated evenly. Meanwhile, due to the diffusion of elements into the tool material and subsurface layer, the precipitated carbides decreased. This was also responsible for the decreased microhardness of the surface layer (as shown in Figure 6). By virtue of the elements diffused from the surface layer, more dispersed carbides precipitated in the subsurface layer, and this improved the microhardness of the subsurface layer (as shown in Figure 6).

#### 3.1.4. Surface Residual Stress

**Figure 10:**
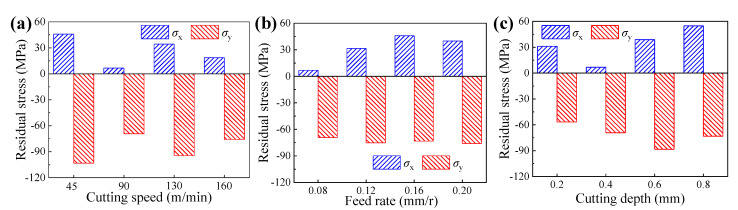
Surface residual stresses of samples obtained under different cutting parameters: (**a**) cutting speed, (**b**) feed rate, and (**c**) cutting depth.

Figure 10 illustrates the surface residual stresses of samples obtained under different cutting parameters, in which the *σ*_x_ and *σ*_y_ mean the residual stresses parallel and perpendicular to the feed rate direction, respectively. It can be found that the residual stresses parallel to the feed rate direction were residual tensile stresses and the residual stresses perpendicular to the feed rate direction were residual compressive stresses. In the feed rate direction, the workpiece material was torn and suffered cutting forces, thus the residual tensile stresses occurred [26]. In the cutting speed direction, the residual compressive stresses were caused by the plastic deformation effect under the interaction of mechanical stress and thermal stress. Meanwhile, for each sample, the residual tensile stress was much smaller than the residual compressive stress, which demonstrates that the dominant stress on the workpiece surface is residual compressive stress, and this can be attributed to the bigger tangential forces than axial forces (as illustrated in Figure 4).

### 3.2. Effect of Hard Turning on Corrosion Resistance

#### 3.2.1. Effect of Hard Turning Process on Corrosion Resistance

**Figure 11:**
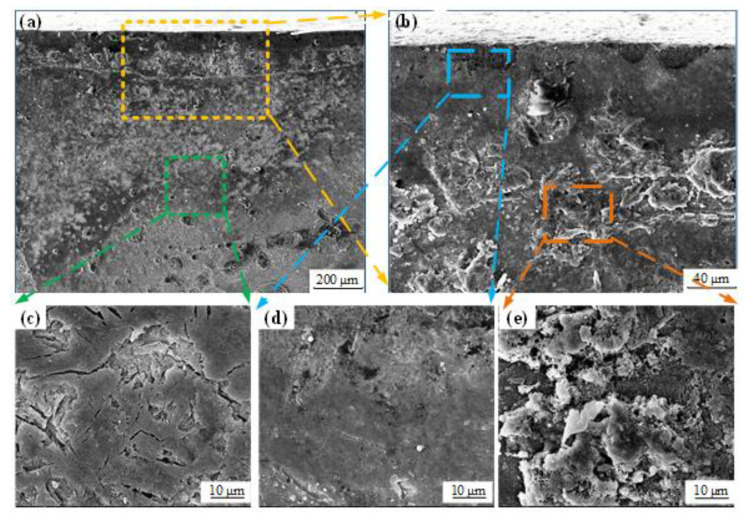
(**a**,**b**) Polarization corrosion morphologies of sample cross section and detail views of (**c**) substrate, (**d**) surface layer, and (**e**) subsurface layer.

Figure 11 shows the polarization corrosion morphologies of the cross section of the sample obtained in Experiment #2. It can be found that the subsurface layer presented severe corrosion morphology with the peeling off of corrosion products and obvious corrosion pits, as shown in Figure 11e. Meanwhile, the substrate material showed local corrosion morphology with some corrosion pits and microcracks, as shown in Figure 11c. In addition, the surface layer had slight corrosion morphology without obvious corrosion pits and microcracks, as shown in Figure 11d. This indicated that the surface layer had the best corrosion resistance, followed by the substrate material and then the subsurface layer. This demonstrates that the hard turning process is feasible to enhance the corrosion resistance of 42CrMo4 steel. By virtue of the uniform carbides and the compact microstructure caused by the residual compressive stress, the surface layer presented good corrosion resistance, whereas the subsurface layer had poor corrosion resistance resulting from the uneven microstructure with coarse carbides. The substrate material had medium corrosion resistance as a result of the moderate microstructure.

#### 3.2.2. Effect of Hard Cutting Parameters on Corrosion Resistance

**Figure 12:**
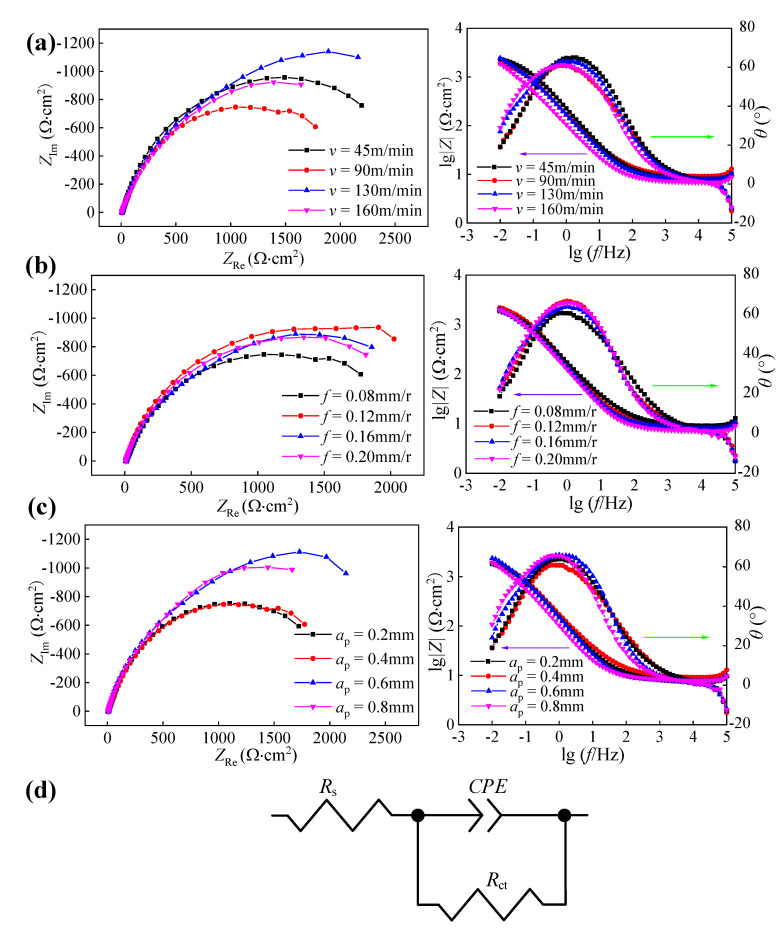
Nyquist plots and Bode plots of machined surfaces for samples machined under different cutting parameters: (**a**) cutting speed, (**b**) feed rate, and (**c**) cutting depth; and (**d**) equivalent circuit model of electrochemical impedance.

To study the influence of hard cutting parameters on the corrosion resistance of 42CrMo4 steel, the electrochemical impedance spectroscopy test and salt spray corrosion test were performed, respectively. The EIS diagrams of machined surfaces for samples obtained under different hard cutting parameters are illustrated in Figure 12a–c.

The Nyquist plot of each sample presented a single capacitive arc, and the bigger diameter of the capacitive arc indicated the better corrosion resistance of machined surfaces. Based on the Nyquist plots, it can be drawn that the surface integrity involved in the hard cutting process had an important impact on the corrosion resistance of machined samples. A single capacitive arc in the Nyquist plot demonstrates that the corrosion reaction was only controlled by the interfacial charge transfer process. The Bode plots of each sample presented only one time constant, and the phase angle was about 65°. The equivalent circuit model is proposed in Figure 12d, in which *R*_s_ means the electrolyte resistance, *R*_ct_ means the charge transfer resistance corresponding to the surface integrity, and *CPE* means the constant phase element, respectively.

**Figure 13:**
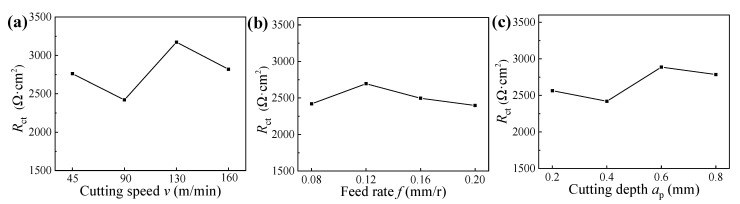
Charge transfer resistances of machined surfaces for samples machined under different cutting parameters: (**a**) cutting speed, (**b**) feed rate, and (**c**) cutting depth.

**Figure 14:**
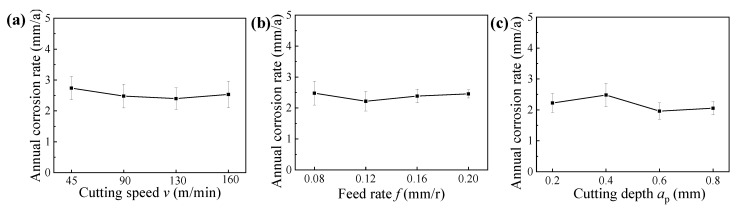
Annual corrosion rates of samples machined under different cutting parameters: (**a**) cutting speed, (**b**) feed rate, and (**c**) cutting depth.

The charge transfer resistances of machined surfaces for samples obtained under different hard cutting parameters are illustrated in Figure 13. It can be found that the values of *R*_ct_ were sensitive to the hard cutting parameters, suggesting that the corrosion resistance of machined samples was deeply dependent on the surface integrity [17]. To further analyze the connection between hard cutting parameters and corrosion resistance of 42CrMo4 steels, the annual corrosion rates of samples machined under different hard cutting parameters were also studied, and the annual corrosion rates obtained after 100 h salt spray corrosion test are presented in Figure 14.

As shown in Figure 3a, Figure 6a, and Figure 10a, respectively, the cutting speed had a smaller influence on the surface roughness, whereas it had a bigger influence on the microhardness and residual stress; thus, it can be concluded that the corrosion resistances of samples machined under different cutting speeds were primarily affected by the surface microhardness and residual compressive stress induced by cutting temperatures. Meanwhile, when the cutting speed was 130 m/min, the sample presented the best corrosion resistance with a big charge transfer resistance and a low annual corrosion rate (as verified in Figure 13a and Figure 14a).

Similarly, as presented in Figure 3b, Figure 6b, and Figure 10b, respectively, the feed rate had a bigger effect on the surface roughness, whereas it had a smaller effect on the microhardness and residual stress; thus, it can be concluded that the corrosion resistances of samples machined under different feed rates were primarily affected by the surface roughness. Meanwhile, when the feed rate was 0.12 mm/r, the sample presented the best corrosion resistance with a big charge transfer resistance and a low annual corrosion rate (as verified in Figure 13b and Figure 14b).

Furthermore, as illustrated in Figure 3c, Figure 6c, and Figure 10c, respectively, the cutting depth had a bigger influence on the surface residual stress, whereas it had a smaller influence on the surface roughness and microhardness; thus, it can be concluded that the corrosion resistances of samples machined under different cutting depths were primarily affected by the surface residual compressive stress induced by the cutting forces and cutting temperatures. Meanwhile, when the cutting depth was 0.6 mm, the sample presented the best corrosion resistance with a big charge transfer resistance and a low annual corrosion rate (as verified in Figure 13c and Figure 14c).

### 3.3. Effect of Hard Cutting Surface Integrity on Corrosion Resistance

To verify the above conclusions and further analyze the effect of surface integrity on the corrosion resistance of 42CrMo4 steel, the potentiodynamic polarization curve test was also conducted, and the data were analyzed combined with the EIS data.

**Figure 15:**
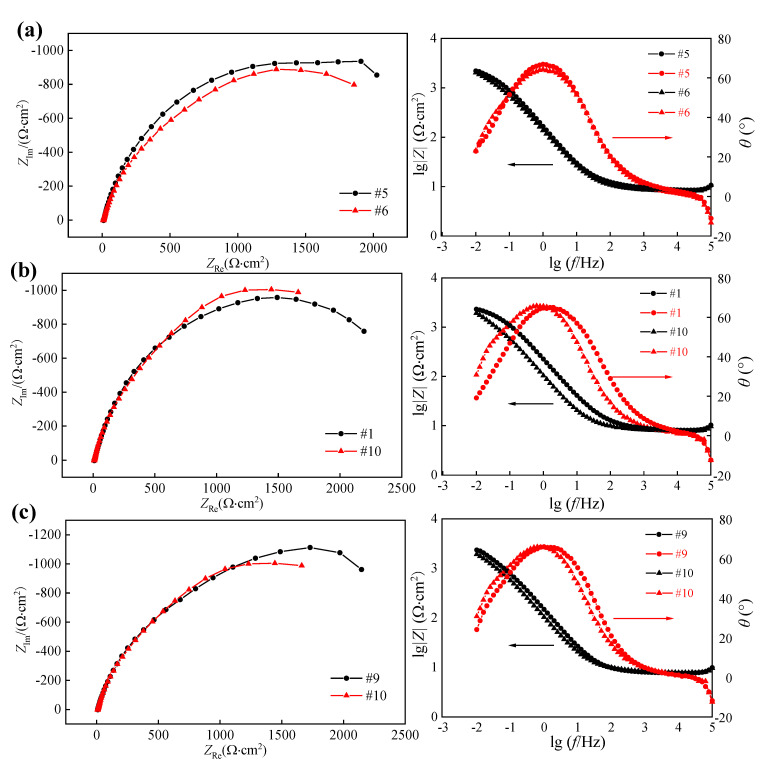
Nyquist plots and Bode plots of samples obtained in comparative experiments: (**a**) #5 and #6, (**b**) #1 and #10, and (**c**) #9 and #10.

**Figure 16:**
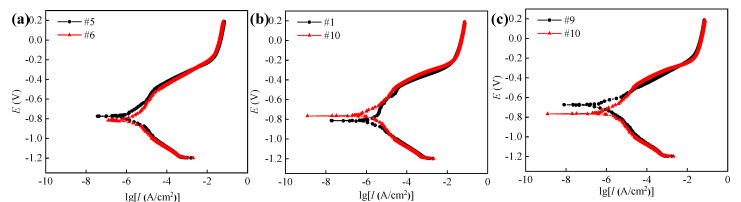
Polarization curves of samples obtained in comparative experiments: (**a**) #5 and #6, (**b**) #1 and #10, and (**c**) #9 and #10.

Table 4 shows the data of surface integrity obtained in the above hard cutting experiment, and it can be found that the samples obtained in Experiments #5 and #6 presented different surface roughness and similar surface microhardness and residual compressive stress. Thus, the samples obtained in Experiments #5 and #6 were used to comparatively study the influence of surface roughness on the corrosion resistance of 42CrMo4 steel. For the same reason, the samples obtained in Experiments #1 and #10 were used to comparatively study the influence of surface microhardness on the corrosion resistance of 42CrMo4 steel, and the samples obtained in Experiments #9 and #10 were used to comparatively study the influence of surface residual compressive stress on the corrosion resistance of 42CrMo4 steel, respectively. The EIS diagrams and polarization curves of machined sample surfaces are displayed in Figure 15 and Figure 16, and the corresponding fitted data are summarized in Table 5 and Table 6, respectively. It can be found that the different samples presented different electrochemical characteristics as a result of the various surface integrity.

Under the similar surface microhardness and residual compressive stress, and bigger surface roughness, the sample obtained in Experiment #6 presented the smaller *R*_ct_ and *E*_corr_, whereas it had bigger *I*_corr_ than those of the sample obtained in Experiment #5. This demonstrates that the surface roughness of the machined sample can affect the corrosion resistance of 42CrMo4 steel, and the bigger the surface roughness is, the worse the corrosion resistance is. It can be attributed to the increased corrosion rate of the rougher machined surface caused by the increased contact area with corrosive medium [27], and the pitting corrosion resulting from more defects, respectively [28].

**Figure 17:**
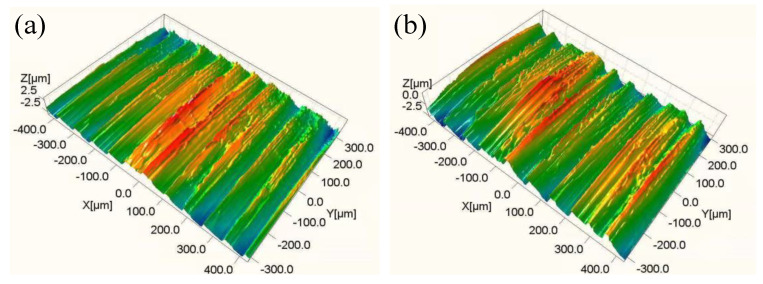
3D morphologies of samples obtained in (**a**) Experiment #5 and (**b**) Experiment #6.

**Figure 18:**
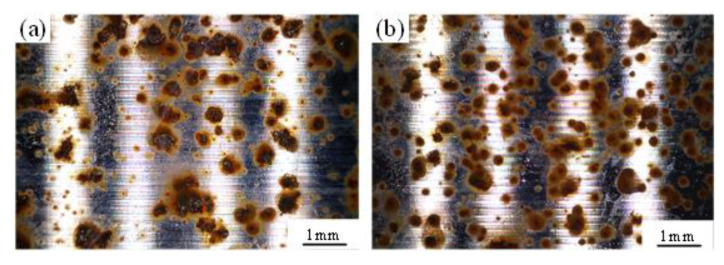
Morphologies of samples obtained in (**a**) Experiment #5 and (**b**) Experiment #6 under salt spray corrosion for 30 min.

Figure 17 show the 3D hard cutting morphologies of samples obtained in Experiments #5 and #6. It can be seen that the sample obtained in Experiment #6 presented a rougher machined surface than those of the sample obtained in Experiment #5, and this was consistent with the obtained values of *n* (dispersion index of *CPE*) in Table 5, the smaller the value of which is, the rougher the surface of sample electrode is. Figure 18 shows the salt spray corrosion morphologies of samples obtained in Experiments #5 and #6. It can be seen that the sample obtained in Experiment #6 presented more corrosion pits than those of the sample obtained in Experiment #5, and the corrosion area ratio was 40% and 36%, respectively. The experimental phenomenon verifies the correctness of the above conclusion.

Under the similar residual compressive stress, bigger surface roughness, and smaller surface microhardness, the sample obtained in the Experiment #10 presented a better corrosion resistance with the bigger *R*_ct_ and *E*_corr_, whereas it had smaller *I*_corr_ than those of the sample obtained in Experiment #1. This demonstrates that the smaller surface microhardness is beneficial for the corrosion resistance of 42CrMo4 steel. It can be ascribed to the microstructure of the surface layer, which presented decreased microhardness and increased corrosion resistance.

Under a similar surface roughness and microhardness, and bigger surface residual compressive stress, the sample obtained in Experiment #9 presented a better corrosion resistance with a bigger *R*_ct_ and *E*_corr_, whereas it had a smaller *I*_corr_ than that of the sample obtained in Experiment #10. This demonstrates that the bigger surface residual compressive stress can improve the corrosion resistance of 42CrMo4 steel. It can be ascribed to the inhibition effects of residual compressive stress on the pitting corrosion and crack growth of 42CrMo4 steel [29,30].

## 4. Conclusions

In this research, the effects of hard turning on surface characteristics and corrosion behaviours were studied. The following conclusions have been obtained:

(1) Hard turning can obtain a surface roughness lower than *Ra* 0.8 μm. The microhardness of the surface layer was lower than that of the substrate due to the softening effect.
(2) Due to the different precipitation behaviours of carbides under the high cutting temperature, the microstructure of the surface layer was fine and uniform, whereas that of the subsurface layer was coarse and uneven. The dominant surface residual stress was residual compressive stress.
(3) The hard turning process is feasible to strengthen the corrosion resistance of 42CrMo4 steel. The good corrosion resistance of the surface layer can be ascribed to the uniform carbides and the compact microstructure caused by the compressive residual stress.
(4) The cutting speeds affected the corrosion resistances of machined samples mainly by the surface microhardness and residual compressive stress induced by cutting temperatures. The feed rates affected the corrosion resistances of machined samples mainly by the surface roughness. The cutting depths affected the corrosion resistances of machined samples mainly by the surface residual stress induced by the cutting forces and cutting temperatures.
(5) By virtue of the decreased contact area with a corrosive medium, the good microstructure of surface layer, and the inhibition effects of residual compressive stress on pitting corrosion and crack growth, the surface integrity with smaller surface roughness and microhardness and bigger surface residual compressive stress was beneficial for the corrosion resistance of 42CrMo4 steel.

This work can guide the practical production and application of hard cutting in strengthening the surface integrity and corrosion resistance of 42CrMo4 high-strength steel. The proposed methodology can be used as a reference for other hardened steels. Furthermore, considering the limitation of hard machining only used for hardened steels, other machining technologies, such as ultrasonic surface rolling process, can be used to strengthen the surface integrity and corrosion resistance of other metal materials.

## Figures and Tables

**Table 1:** Chemical compositions of 42CrMo4 steel (wt.%).

C	Si	Mn	P	Cu	Ni	Cr	Mo	Fe
0.41	0.22	0.65	0.01	0.03	0.02	1.04	0.17	Balance

**Table 2:** Tool geometric parameters.

Cutting Edge Angle *κ*_r_	Relief Angle *α*_o_	Rake Angle *γ*_o_	Inclination Angle *λ*_s_	Nose Radiu *r_ε_*
95°	6°	−6°	−6°	1.2 mm

**Table 3:** Experimental design.

Experiment	Cutting Speed (m/min)	Feed Rate (mm/r)	Cutting Depth (mm)
#1	45	0.08	0.4
#2	90	0.08	0.4
#3	130	0.08	0.4
#4	160	0.08	0.4
#5	90	0.12	0.4
#6	90	0.16	0.4
#7	90	0.20	0.4
#8	90	0.08	0.2
#9	90	0.08	0.6
#10	90	0.08	0.8

**Table 4:** Data of surface integrity obtained in cutting experiments.

Experiment	Surface Roughness (μm)	Surface Microhardness (HV0.1)	Surface Residual Compressive Stress (MPa)
#1	0.586	486	81
#2	0.680	471	57
#3	0.579	454	109
#4	0.585	434	70
#5	0.850	459	67
#6	0.963	460	67
#7	2.142	447	55
#8	0.738	467	66
#9	0.756	453	97
#10	0.753	457	82

**Table 5:** Fitted data of electrochemical impedance spectroscopy corresponding to Figure 15.

Experiment	*R*_ct_ (Ω·cm^2^)	*R*_s_ (Ω·cm^2^)	*Y* (Ω^−1^·cm^−2^·s^−*n*^)	*n*
#1	2762	8.448	1.079 × 10^−3^	0.768
#5	2695	9.189	1.434 × 10^−3^	0.785
#6	2494	8.473	1.829 × 10^−3^	0.766
#9	2887	7.893	1.627 × 10^−3^	0.788
#10	2786	8.139	2.379 × 10^−3^	0.776

**Table 6:** Fitted data of polarization curves corresponding to Figure 16.

Experiment	*E*_corr_ (V)	*I*_corr_ (μA·cm^−2^)	*βa* (V·dec^−1^)	|*βc*| (V·dec^−1^)
#1	−0.817	1.778	0.352	0.214
#5	−0.765	1.585	0.294	0.250
#6	−0.815	2.512	0.339	0.239
#9	−0.680	0.708	0.138	0.238
#10	−0.745	1.660	0.272	0.261

## Data Availability

Data are contained within the article.
